# Aspacochioside C from *Asparagus cochinchinensis* attenuates eumelanin synthesis via inhibition of TRP2 expression

**DOI:** 10.1038/s41598-023-41248-5

**Published:** 2023-09-08

**Authors:** Silvia Yunmam, Hae Ran Lee, Seong Min Hong, Ji-Young Kim, Tong Ho Kang, Ai Young Lee, Dae Sik Jang, Sun Yeou Kim

**Affiliations:** 1 College of Pharmacy, Gachon University, 191, Hambakmoero, Yeonsu-gu, Incheon, 21936 Republic of Korea; 2 Institute of Bioresources and Sustainable Development, Imphal, Manipur 795001 India; 3 Department of Biomedical and Pharmaceutical Sciences, Graduate School, Kyung Hee University, Seoul, 02447 Republic of Korea; 4 Department of Oriental Medicine Biotechnology, College of Life Sciences and Graduate School of Biotechnology, Kyung Hee University, Global Campus, Gyeonggi, 17104 Republic of Korea; 5 Department of Dermatology, Graduate School of Medicine, Dongguk University Seoul, Goyang, Republic of Korea; 6 Gachon Institute of Pharmaceutical Science, Gachon University, Yeonsu-gu, Incheon, 21565 Republic of Korea

**Keywords:** Biochemistry, Biomarkers, Diseases

## Abstract

Aspacochioside C (ACC) is a steroidal saponin isolated from *Asparagus cochinchinensis*. Steroidal saponins, such as pseudoprotodioscin and dioscin, are known to inhibit melanogenesis, but the role of ACC in melanogenesis remains unknown. Due to the toxic effect of the commonly used skin whitening agents like arbutin, kojic acid and α-lipoic acid alternative plant products are recentlybeen studied for their anti-hypergmentation effect. This study explores the role of ACC in melanogenesis in both in vivo and in vitro models. Here, we for the first time demonstrate that ACC attenuated α-MSH- and UVB-induced eumelanin production by inhibiting tyrosinase-related protein (TRP)-2 protein expression in both murine B16F10 and human melanoma MNT1 cells. However, ACC had no significant effect on pheomelanin concentration. ACC also decreased the pigmentation density in zebrafish embryos, which indicates that ACC targets TRP2 and inhibits eumelanin synthesis. Our results demonstrate that ACC inhibits TRP2, thereby attenuating eumelanin synthesis both in in vitro and in vivo zebrafish model. Therefore, ACC can potentially be used as an anti-melanogenic agent for both aesthetic and pharmaceutical purposes.

## Introduction

Skin is the largest organ and the most exposed area of the body. It is the first line of defense and reacts to various external stimuli, including ultraviolet radiation (UVR). Melanin protects the skin from harmful UVR exposure and other environmental stressors. A number of factors at the systemic, tissue, cellular and subcellular levels regulate melanin pigementation. Exposure to UVR increases melanin synthesis, causing immediate pigmentation and skin darkening. Chronic exposure to UV also causes photoaging and melanoma^1,2^. Therefore, many studies have focused on the suppression of melanin production. Many of the skin whitening agents like arbutin, kojic acid and α-lipoic acid which are commonly used in the cosmetic industry are toxic and cause side effects like dermatitis, acne, allergy, hypertension etc. In recent years, several natural products have been used for anti-hyperpigmentation and whitening research.

Melanogenesis is the process of melanin synthesis by endogenous melanocytes in specific organelles, called melanosomes. Melanoblasts, the precursor of melanocytes are derived from neural crest cells originating in the neural tubes. They migrate to different parts of the body including skin, hair follicles, eyes, inner ear, bones, heart, and brain where they develop into melanocytes^3^. The cutaneous melanocytes produced melanin which is made to filter ultraviolet radiation (UVR) to prevent DNA degeneration or oxidation that acts on the skin. Futhermore, melanin prevents melanomagenesis^4^. This melanin is produced by melanogenic factors, such as tyrosinase, tyrosinase-related protein (TRP)-1, TRP-2, and melanocyte-inducing transcription factor (MITF), are associated with melanin synthesis^3^. Mainly, the melanin synthesis is divided two types of melanin: eumelanin and pheomelanin by transformed L-tyrosine to L-DOPA through oxidoreduction reactions^5,6^. In further progress, dopaquinone is oxidized to L-DOPA by tyrosinase, it is common precursor to both pheomelanin and eumelanin^7^. Dopaquinone reacts with cysteine to produce sulfur containing benzothiazine melanin and benzothiazole melanin derivaties which subsequently produces reddish yellow pigment, pheomelanin^8^. There is spontaneous cyclization of dopaquinone to leukodopachrome when intramelasomal cysteine is delepleted. Leukodopachrome reacts with unchanged dopaquinone to form orange dopachrome. Decarboxylation of dopachrome give rise to 5,6-dihydroxyindole (DHI) which further polymerizes to form brownish-black eumelanin. TRP2 possesses dopachrometautomerase activity that tautomerises dopachrome to 5,6-dihydroxyindole-2-carboxylic acid (DHICA), andTRP1oxidizes DHICA to moderately soluble lighter brown eumelanin^9,10^. A single melanocyte is surrounded by approximately 40 keratinocytes. Melanin synthesized in the melanocytes are transferred to the keratinocytes where the pigments are retained providing colors to the skin and hair^11^. Keratinocytealso regulates eumelanin production through the release of various cytokines and chemokines after UVR exposure. UVR induced keratinocytes to secrete α-MSH and adenocorticotopic hormone which signals melanocytes to produce melanin^12^. Eumelanin has more photo-protecting properties than pheomelanin. Eumelanin has anti-oxidant activity and scavenges reactive oxygen species (ROS) while pheomelanin generates ROS through UV dependent or independent pathways^13^. Increased ROS production during pheomelanin synthesis is considered to be one of the reasons by which pheomelanin promotes carcinogenesis^12^.

*Asparagus cochinchinensis* (Loureio) Merrill (*Liliaceae*) is a perennial herb distributed in Eastern Asia that is used in traditional medicine to treat heart diseases, lung cancer, renal failure, and fever^14^. The roots of *A. cochinchinensis* consist of a range of steroidal saponins, namely protodioscin, methyl protodioscin, aspacochioside A, aspacochioside C (ACC), and 15-hydroxy pseudoprotodioscin^15^. These compoundsare used as anti-aging^14,16^, anti-diabetic, anti-tumor^17,18^, and anti-neurodegenerative^19,20^ agents. Previously, *A. cochinchinensis* extract fermented using *Aspergillus oryzae* was shown to have anti-tyrosinase and anti-melanogenic effects on human melanocytes; however, the exact anti-melanogenesis mechanism of unfermented *A. cochinchinensis* root extracts has not yet been investigated^21^. In the present study, we demonstrated for the first time that ACC isolated from *A. cochinchinensis* has anti-melanogenic effects as it inhibits eumelanin synthesis in both in vitro melanocytes and in vivo zebrafish models.

## Results

### Effect of ACC on melanin pigmentation in zebrafish

**Figure 1:**
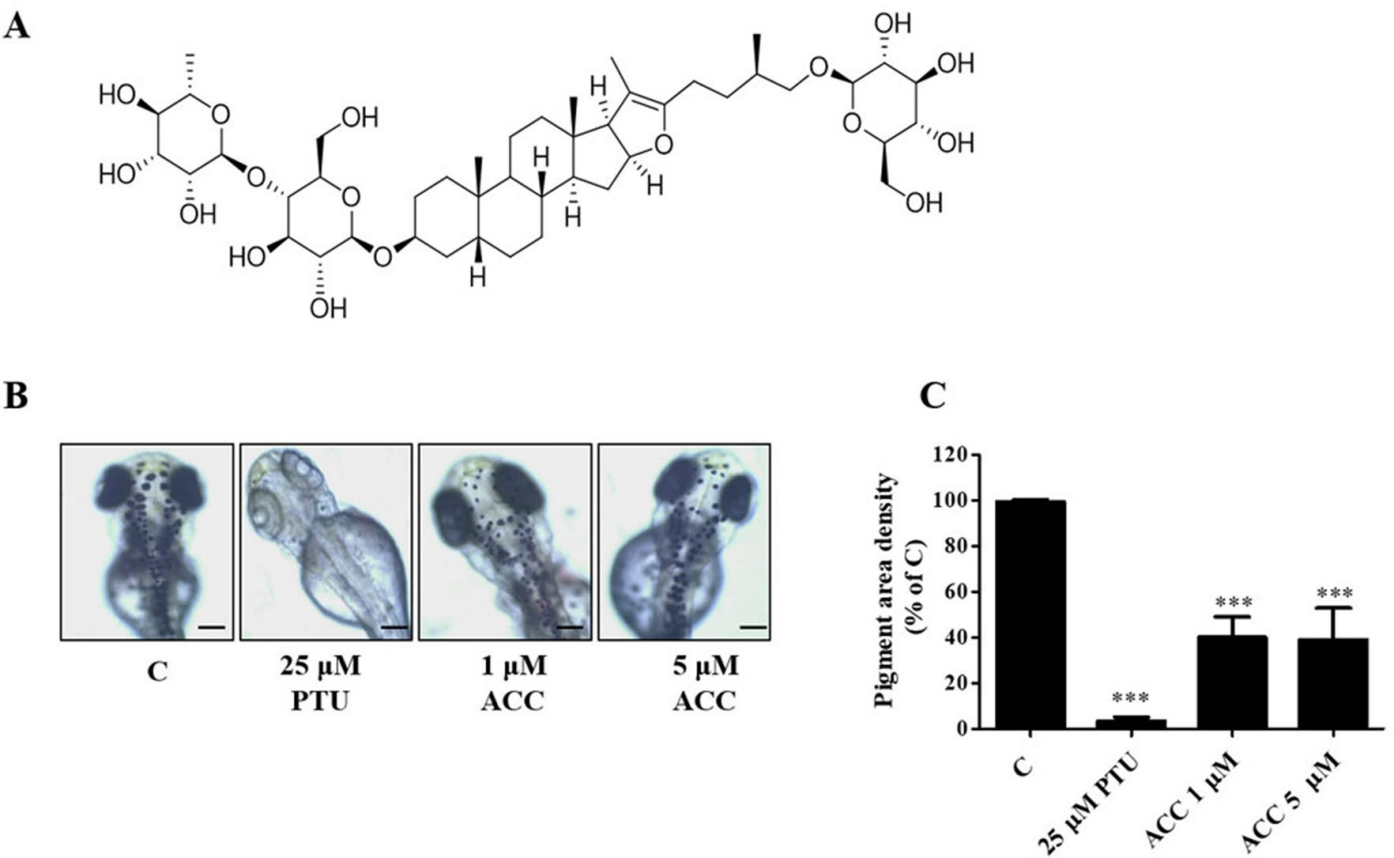
Effect of aspacochioside C (ACC) on zebrafish pigmentation. (**A**) Chemical structure of ACC. Zebrafish embryos were treated with or without ACC (1 or 5 μM) and phenylthiourea (PTU, 25 μM). PTU was used as a positive control. (**B**) ACC inhibited zebrafish pigmentation. Pigmentation in zebrafish embryos was observed under a IX7 microscope at 72 hpf. Scale bar: 0.5 mm. (**C**) Pigmentation area density was determined using the ImageJ software (n = 5). The values represent the mean ± standard error of the mean (SEM) of three independent experiments. ^***^p < 0.001 versus C.

Cell-free mushroom tyrosinase activity of the hot water extract of *A. cochinchinensis* and five steroidal saponins, including ACC, was determined in both murine B16F10 and human melanoma MNT1 cells. The hot water extract of *A. cochinchinensis* and five steroidal saponins, including ACC, had no significant effects on the mushroom tyrosinase activity. Among them, only ACC showed about 15–20% tyrosinase inhibition activity (Supplementary Fig. 1). Figure 1A shows the chemical structure of ACC. To determine the anti-pigmentation effect of ACC, an in vivo assay was performed. Zebrafish embryos at 9 hfp (hour post-fertilization) were treated with various concentrations of ACC and observed at 72 hfp. Phenylthiourea (PTU, 25 µM) was used as the positive control. As shown in Fig. 1B, PTU completely inhibited melanogenesis, while 1 or 5 μM ACC significantly inhibited the pigmentation density in developing zebrafish embryos (Fig. 1C). Higher concentrations of ACC were toxic to the zebrafish embryos (results not shown). Embryos are highly sensitive to chemicals and can be particularly toxic at higher dose. In this study, we used Zebrafish embryos at 9 hpf and observed them at 72 hpf. As shown in Fig. 1B, 1 or 5 μM ACC significantly inhibited the pigmentation density in developing zebrafish embryos and the concentration that shows this effect was not toxic. But higher concentrations of 10 μM ACC were toxic to the zebrafish embryos. The toxicity of sample in in vivo can provide information on sample distribution in the organ and possible interaction of sample with non-target organs. And also it can occur as a result of over dose of the medication. So higher toxicity of ACC may occur as an adverse reaction of overdose. And as ACC did not show any toxicity at the melanocytes-derived cells, we think that we can overcome this problem with skin application rather than oral administration. We also found that there was no complete loss of pigmentation, which suggests that ACC may not completely inhibit melanin synthesis. To further study the effect of ACC on melanogenesis, in vitro studies were carried out.

### Effects of ACC on the tyrosinase activity and melanin production in melanocytes

**Figure 2:**
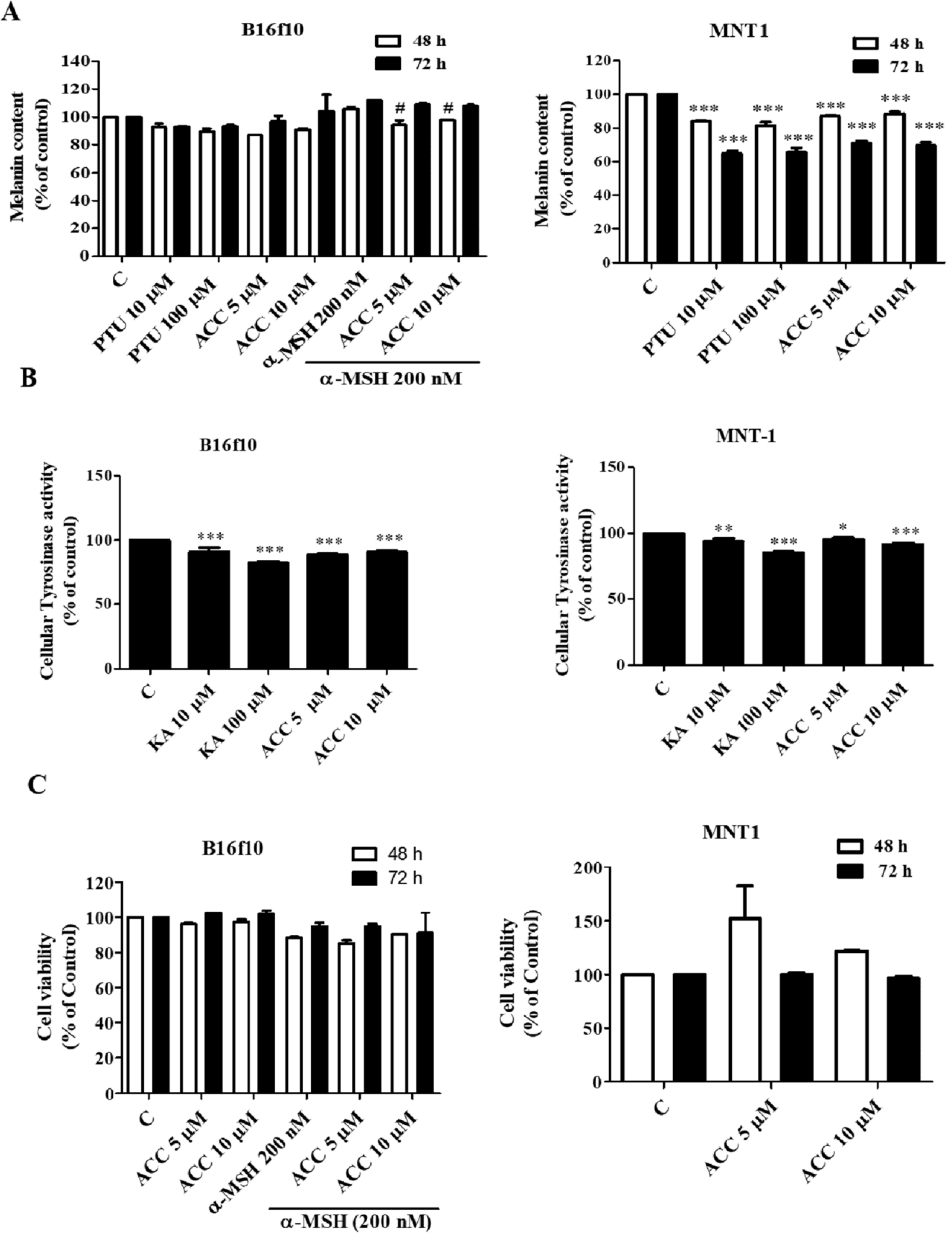
Effect of aspacochioside C (ACC) on melanin production. B16F10 cells were treated with or without ACC (5 or 10 μM) and 200 nM alpha-melanocyte-stimulating hormone (α-MSH), while MNT1 cells were treated with or without ACC (5 or 10 μM) for 48 and 72 h. (**A**) ACC inhibited, while α-MSH induced melanin production in MNT1cells. PTU was used as a positive control. (**B**) ACC decreased the cellular tyrosinase activity in B16F10 and MNT1cells. Kojic acid (KA) was used as a positive control. (**C**) ACC had no cytotoxic effects on B16F10 and MNT1 cells. The values represent the mean ± (SD) of three independent experiments. ^*^p < 0.05, ^***^p < 0.001 versus C; ^#^*p* < 0.05 versus α-MSH.

Melanin synthesis and cellular tyrosinase activity were evaluated in ACC-treated B16F10 and MNT1 cells. ACC (5 and 10 μM) inhibited melanin production in a time-dependent manner. In B16F10 cells, ACC inhibited around 10% of α-MSH induced melanin content. Around 20% of melanin content was inhibited at 48 h, while around 40% of melanin content was inhibited at 72 h in MNT1 cells (Fig. 2A). ACC also inhibited approximately 20% of the cellular tyrosinase activity in B16F10 and MNT1 cells (Fig. 2B). In addition, a 3-(4,5-dimethylthiazol-2-yl)-2,5-diphenyl tetrazolium bromide assay was performed to determine the cytotoxic effects of ACC on B16F10 and MNT1 cells. As shown in Fig. 2C, ACC had no cytotoxic effects on B16F10 or MNT1 cells. These results suggest that ACC inhibited melanin synthesis in B16F10 and MNT1 cells.

### Effects of ACC on melanogenesis-related proteins

**Figure 3:**
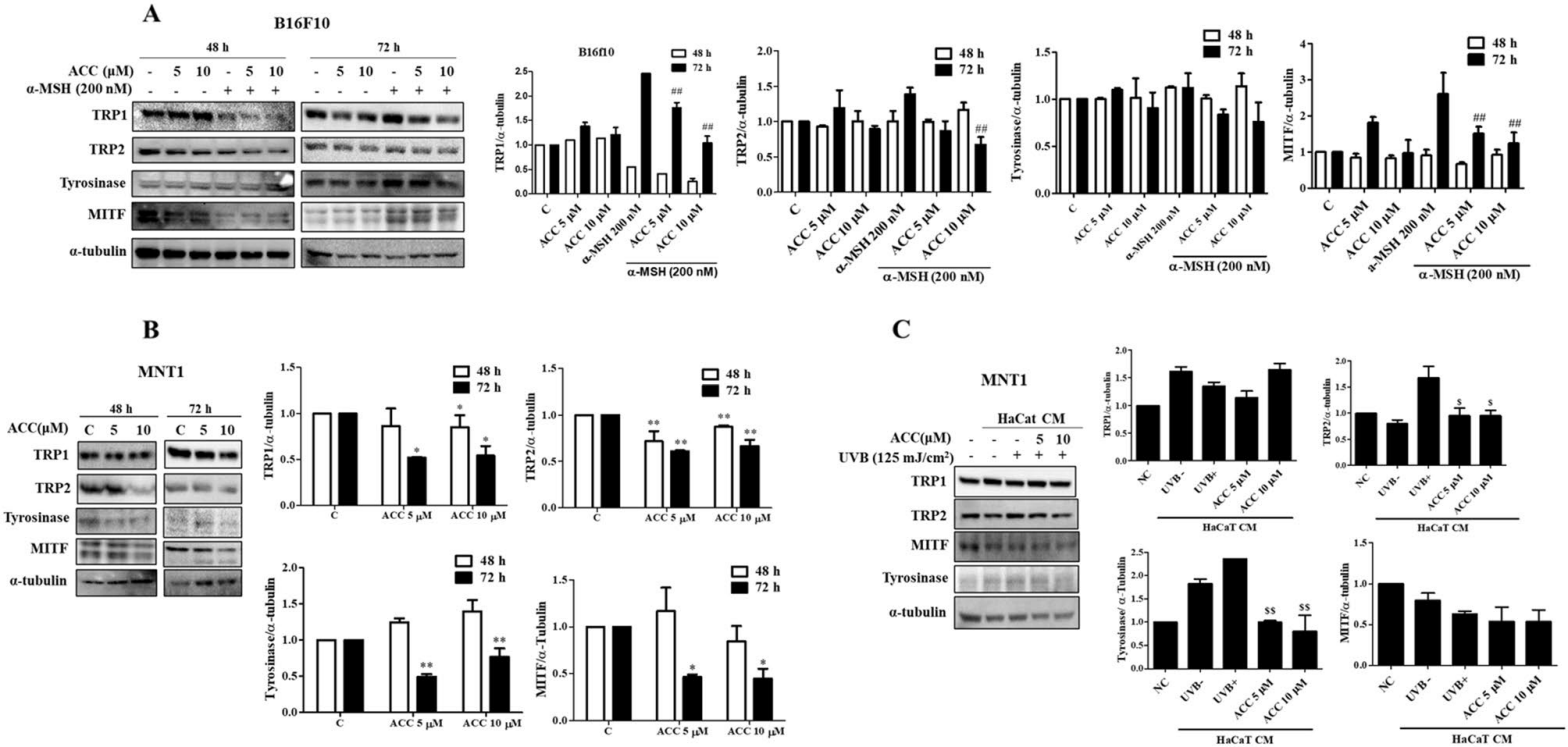
Effects of aspacochioside C (ACC) on melanogenesis-related proteins. (**A**) ACC inhibited α-MSH-induced tyrosinase-related protein (TRP)-2 and melanocyte-inducing transcription factor (MITF) protein expression in B16F10 cells. B16F10 cells were treated with or without ACC (5 or 10 μM) and 200 nM α-MSH for 48 and 72 h. Cells were lysed and proteins were separated according to size via sodium dodecyl sulfate–polyacrylamide gel electrophoresis (SDS-PAGE). (**B**) ACC inhibited TRP1, TRP2, tyrosinase, and MITF protein expression in MNT1 cells. Cells were treated with or without ACC (5 or 10 μM) 48 and 72 h. Cells were lysed and proteins were separated according to size via SDS-PAGE. (**C**) ACC inhibited UVB-induced TRP2 and tyrosinase protein expression in MNT1 cells. HaCaT cells were irradiated with 125 mJ/cm^2^ UVB and treated with or without ACC (5 or 10 μM). After 24 h, conditioned media (CM) of HaCaT cells were collected and used to treat MNT1 cells. After 48 h, proteins were collected and separated according to size via SDS-PAGE. NC: MNT1 cells grown in 2% fetal bovine serum (FBS) containing Dulbecco's Modified Eagle’s Medium (DMEM), UVB: MNT1 cells grown in HaCaT CM, UVB^+^: MNT1 cells grown in UVB irradiated HaCaT CM. The values represent the mean ± SD of three independent experiments. ^*^p < 0.05, ^***^p < 0.001 versus C; ^##^*p *< 0.01 versus α-MSH; ^$$^*p* << 0.01 versus UVB^+^.

To study the relationship between melanin synthesis inhibition, western blotting analysis of melanogenesis-related proteins was performed in ACC-treated B16F10 and MNT1 cells. ACC significantly inhibited α-MSH-induced TRP2 and MITF protein expression levels in B16F10 cells at 72 h, whereas at 48 h, there was no significant inhibion of melanogenesis related proteins. Similarly, ACC significantly inhibited TRP1, TRP2, MITF, and tyrosinase protein expression levels at 72 h, while at 48 h, ACC only significantly inhibited TRP1 and TRP2 protein expression levels in MNT1 cells (Fig. 3A,B). Treatment with 5 and 10 μM ACC showed similar inhibition of melanogenesis in both B16F10 and MNT1 cells, indicating that there is no concentration-dependent inhibition of melanogenesis. As sun exposure is a major cause of hyperpigmentation and UVB-irradiated keratinocytes increase α-MSH production, which activates melanin production by melanocytes, we exposed HaCaT cells to 125 mJ/cm^2^ UVB and then treated with ACC. The conditioned media (CM) of HaCaT cells irradiated with UVB and treated with ACC were collected and used to treat MNT1 cells. ACC significantly inhibited UVB-induced TRP2 and tyrosinase protein expression levels in MNT1 cells (Fig. 3C), suggesting that ACC also inhibited UVB-induced melanogenesis. ACC significantly inhibited α-MSH-induced TRP1 gene expression levels in B16F10 and MNT1 cells, but there were no significant changes in TRP2 gene expression levels in both B16F10 and MNT1 cells (Supplementary Fig. 2). Similarly, ACC had no significant effect on UVB-induced TRP1 and TRP2 expression levels (Supplementary Fig. 3). Taken together, our results suggest that ACC inhibits α-MSH- and UVB-induced TRP2 protein expression levels in B16F10 and MNT1 cells.

### Effect of ACC on eumelanin and pheomelanin synthesis

**Figure 4:**
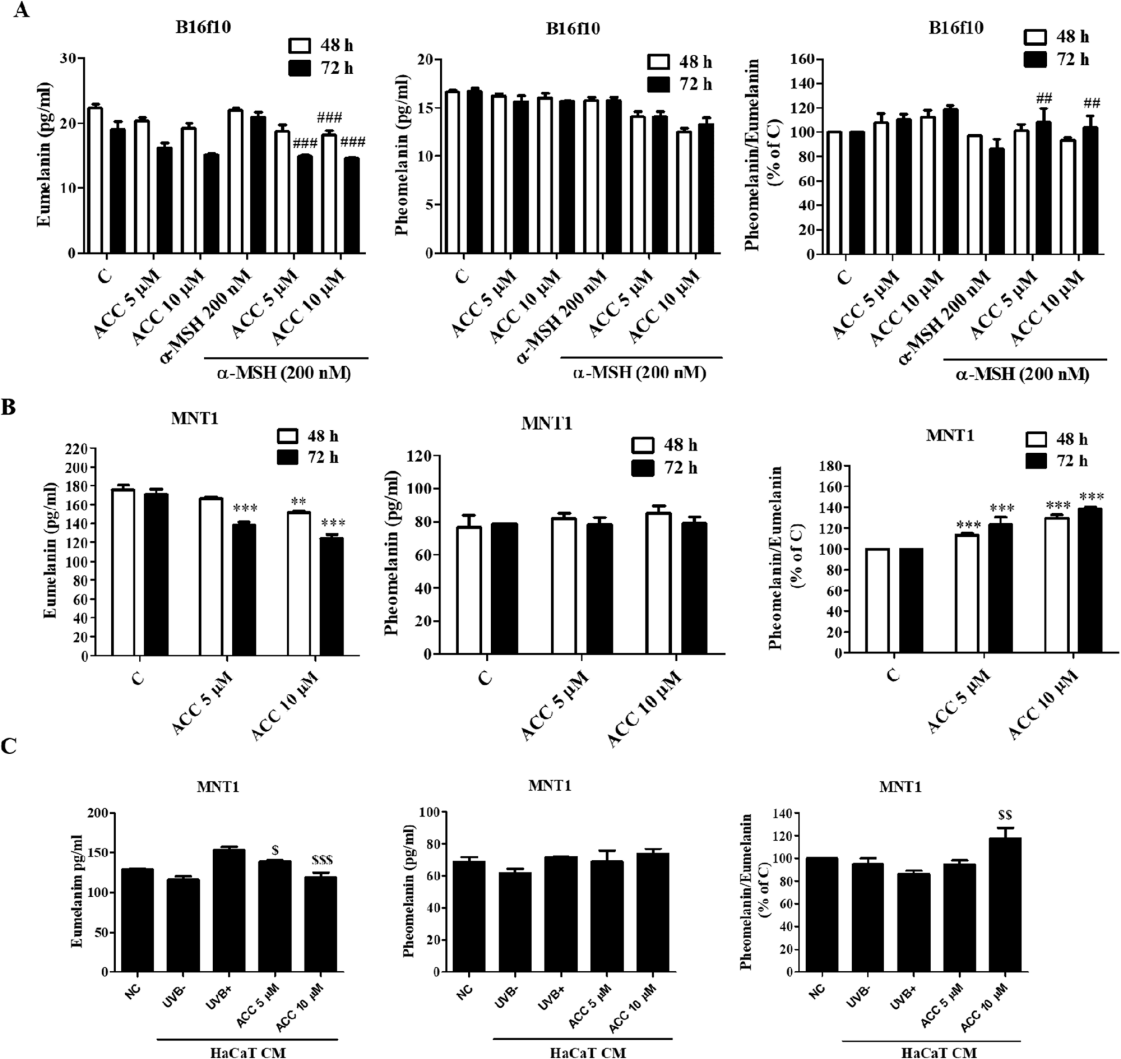
Effect of aspacochioside C (ACC) on eumelanin and pheomelanin production. Cells were treated with ACC as described. Eumelanin and pheomelanin concentrations were measured using Eumelanin and Pheomelanin enzyme-linked immunosorbent assay (ELISA) kit, according to the manufacturer’s protocol. (**A**) ACC inhibited α-MSH-induced eumelanin production in B16F10 cells, with no significant change in pheomelanin production. (**B**) ACC inhibited eumelanin production in MNT1 cells, with no significant change in pheomelanin production. (**C**) ACC inhibited UVB-induced eumelanin production in MNT1 cells, with no significant change in pheomelanin production. NC: MNT1 cells grown in 2% FBS containing DMEM, UVB^−^: MNT1 cells grown in HaCaT CM, UVB^+^: MNT1 cells grown in UVB irradiated HaCaT CM. The values represent the mean ± SD of three independent experiments. ^***^p < 0.001 versus C; ^##^*p* < 0.01, ^###^*p* < 0.001 versus α-MSH; ^$^*p* < 0.05, ^$$^*p* < 0.01, ^$$$^*p* < 0.001 versus UVB^−^.

**Figure 5:**
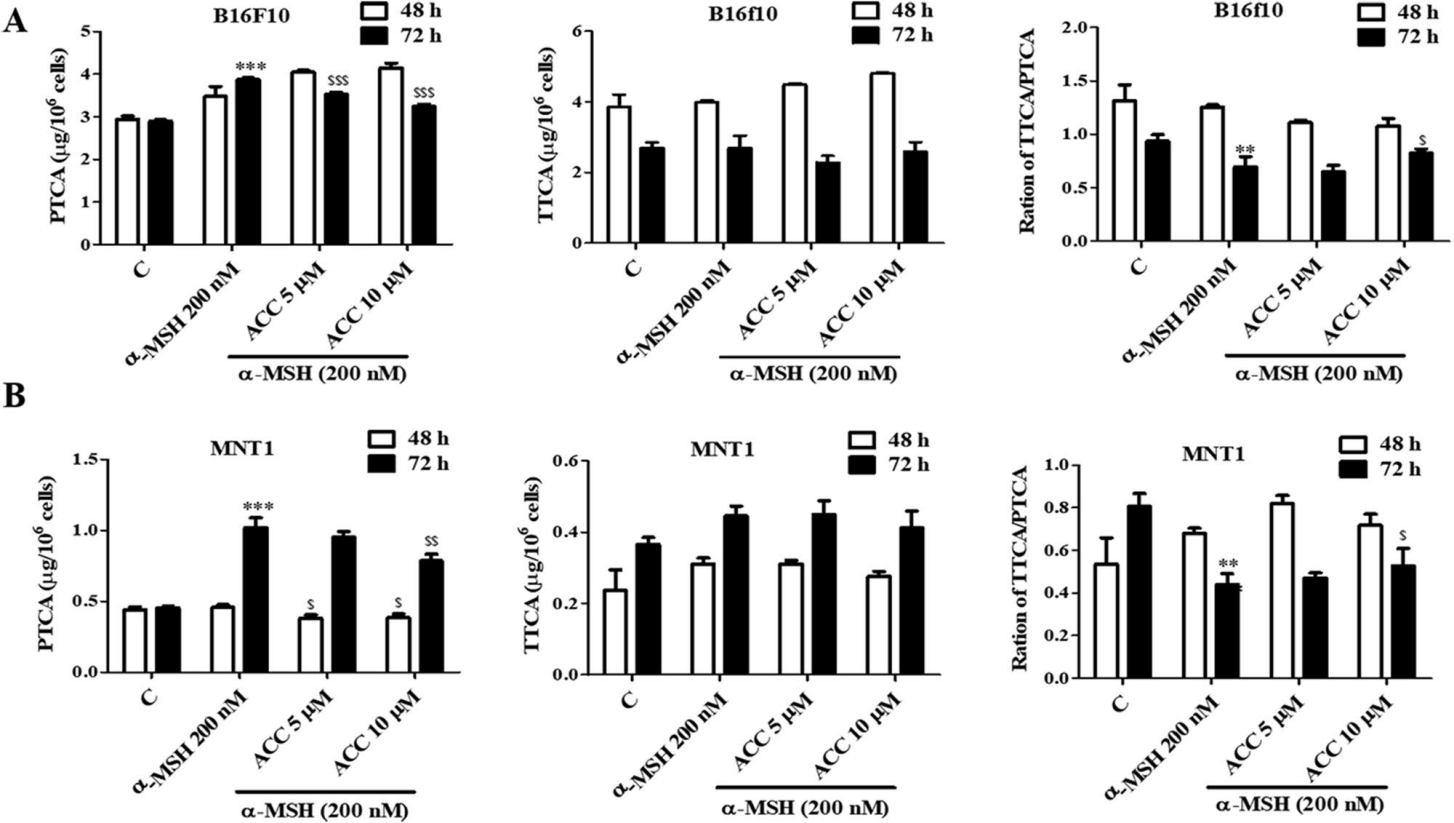
Effect of aspacochioside C (ACC) on ratio of pheomelanin (TTCA)/eumelanin (PTCA) production. Cells were treated with ACC as described. The ratio of pheomelanin (TTCA)/eumelanin (PTCA) was measured using HPLC analysis. (**A**) ACC inhibited α-MSH-induced eumelanin (PTCA) production in B16F10 cells, with no significant change in pheomelanin (TTCA) production. (**B**) ACC inhibited α-MSH-induced eumelanin (PTCA) production in MNT1 cells, no significant change in pheomelanin (TTCA) production. The values represent the mean ± SD of three independent experiments. ^***^p < 0.001 versus C; ^##^*p* < 0.01, ^$^*p* < 0.05, ^$$^*p* < 0.01, ^$$$^*p* < 0.001 versus α-MSH.

As melanin consists of eumelanin and pheomelanin, the effect of ACC on eumelanin and pheomelanin synthesis was determined in both B16F10 and MNT1 cells using eumelanin and pheomelanin ELISA kits. ACC significantly suppressed α-MSH-induced eumelanin content in B16F10 (Fig. 4A) and MNT1 cells (Fig. 4B). Moreover, there was no significant change in the pheomelanin content in either B16F10 or MNT1 cells. ACC also decreased UVB-induced eumelanin production in MNT1 cells (Fig. 4C). Concomitant with TRP2 protein expression, the effect of ACC on eumelanogenesis was time-dependent, not concentration-dependent. As shown in Fig. 4, there was around 20% increase in the pheomelanin/eumelanin ratio in α-MSH-induced B16F10 cells, 20–40% increase in MNT1 cells, and 20% increase in UVB-induced MNT1 cells. In addition, eumelanin marker PTCA (pyrrole-2,3,5-tricarboxylic acid) and pheomelanin marker TTCA (thiazole-2,4,5-tricarboxylic acid) were quantitatively analysed in α-MSH-induced B16F10 and MNT1 cells using HPLC analysis. ACC significantly suppressed α-MSH-induced PTCA content in B16F10 (Fig. 5A) and MNT1 cells (Fig. 5B). Meanwhile, the content of TTCA was not changed in both cell lines but ACC significantly enhanced the ratio of TTCA/PTCA in α-MSH-induced B16F10 and MNT1 cells (Fig. 5 and Supplementary Fig. 4). Taken together, our results suggest that ACC inhibits eumelanin production by regulating TRP2 expression levels in B16F10 and MNT1 cells.

## Discussion

Under normal conditions, melanin protects the skin from harmful environmental factors, such as UVR. Skin hyperpigmentation is a result of chronic UV exposure, and many diseases, such as melasma, Addison’s disease, post-inflammatory hyperpigmentation, and melanoma, are associated with hyperpigmentation. In the cosmetic industry, many tyrosinase inhibitors, such as arbutin, kojic acid, and ascorbic acid, are commonly used as skin whitening agents. However, these anti-melanogenic agents are highly toxic^22^. Therefore, more effective and safe treatment methods are required for dermatological and aesthetic purposes. Although steroidal saponins, such as pseudoprotodioscin isolated from fenugreek^23^ and dioscin derived from *Solanum melongena*^24^, have been shown to attenuate α-MSH-induced melanogenesis in B16F10 cells, the role of ACC, a steroidal saponin isolated from *A. cochinchinensis*, in melanogenesis has not yet been studied. In the present study, we demonstrated for the first time that ACC inhibited eumelanin synthesis in murine B16F10 and human MNT1 cells. In addition, ACC inhibited the pigmentation density in zebrafish embryos, indicating that ACC has anti-melanogenic properties.

Both eumelanin and pheomelanin are derived from the common precursor, dopaquinone, which is formed by the oxidation of L-tyrosine by tyrosinase. Dopaquinone enters the pheomelanin pathway when it reacts with L-cysteine to form cysteinyldopa. In the absence of thiol compounds, amino groups are added to dopaquinone to produce cyclodopa, which is then oxidized to dopachrome, which enters the eumelanin synthesis pathway. The trapping of dopaquinone by L-cysteine increases pheomelanin synthesis, while decreasing eumelanin synthesis^25^. In addition to tyrosinase, TRP1 and TPR2 modulate eumelanogenesis^26^. TRP2 is involved in the modification of pigment color, and its inhibition leads to the modification of color rather than loss of pigmentation. TRP2 expression determines the color coat of sheep^27^ and TRP2 knockout mice have less melanin pigmentation than normal mice^28^. TRP2 plays major roles in the early stages of melanin formation, growth, survival, and melanocyte function^29^**.** Recently, Lee et al.^30^ demonstrated that TRP2 downregulation inhibits melanin synthesis in B16F10 cells and showed that targeting TRP2 may be a possible anti-melanogenesis therapeutic strategy. In the present study, we found that ACC increased around 20% of the pheomelanin/eumelanin ratio in α-MSH-induced B16F10 cells, along with 20–40% increase in MNT1 cells and 20% increase in UVB-induced MNT1 cells, thus inhibiting eumelanin production. In addition, ACC significantly decreased PTCA content which is a eumelanin marker while TTCA which is a pheomelanin marker did not change in both MNT1 and B16F10 cells. Moreover, ACC significantly attenuated α-MSH- and UVB-induced TRP2 and tyrosinase expression levels in B16F10 and MNT1 cells, suggesting that ACC inhibits eumelanin production by regulating TRP2 expression. Interestingly, these results are consistent with the in vivo results, as ACC inhibited the pigmentation density in zebrafish embryos, without the complete loss of pigmentation, as TRP2 inhibition does not lead to complete loss of pigmentation. ACC partially inhibits tyrosinase activity, which potentially reduces dopaquinone synthesis and dopachrome formation by inhibiting TRP2 activity, which leads to the inhibition of eumelanin synthesis. Therefore, ACC inhibits eumelanin synthesis by reducing and regulating TRP2 activity, with no significant change in pheomelanin synthesis. As darker skin contains more eumelanin than lighter skin, skin care products that inhibit eumelanin production can be used for darker skin tones, thus personalizing the skin care approach. TRP2 protects melanoma from UVB-induced apoptosis^31^ and is a marker of amelanotic and melanotic melanoma^32^. Many studies have reported the importance of melanin synthesis in advanced melanoma therapy. Brożyna et al.^4^ have shown that inhibition of tyrosinase and melanin production in melanoma cells enhanced the effect of gamma rays in inhibiting melanoma cell growth. In another study the effect of *Coriolus versicolor* a Chinese fungus was found to be more susceptible in inducing cell death after depigmentation of melanoma cells^33^. Lee et al.^30^ also demonstrated that *N*-(3,5-dimethylphenyl)-3-methoxybenzamide inhibits cell proliferation in B16F10 melanoma cells by inhibiting TRP2 protein expression, and thus they concluded that targeting TRP2 may also be a useful strategy for melanoma treatment. Thus, TRP2 inhibitors can be used for both aesthetic and pharmaceutical applications. In future studies, we will determine if ACC targeting TRP2 will have anti-melanoma effect on melanoma cells.

In conclusion, the present study demonstrated that ACC markedly inhibited eumelanin synthesis via the degradation of TRP2. Our results revealed that ACC attenuated α-MSH- and UVB-induced eumelanin production by regulating TRP2 protein expression in both murine B16F10 and human MNT1 melanoma cells. Additionally, ACC had the same effect on zebrafish embryos, as it inhibited pigmentation. A previous study showed that steroidal saponins have anti-melanogenic effects; however, for the first time, we have demonstrated the anti-eumelanogenic effect of ACC in both α-MSH- and UVB-induced melanocytes. Based on these results, we suggest that ACC can potentially be used as an anti-melanogenic agent for both aesthetic and pharmaceutical purposes.

## Material and methods

### Chemical and reagents

Dulbecco’s modified Eagle’s medium (DMEM) was purchased from HyClone (Logan, UT, USA). Fetal bovine serum (FBS) and 1X phosphate-buffered saline (PBS) were purchased from GIBCO (Thermo Fisher Scientific Inc., Waltham, MA, USA). Antibodies against tyrosinase, TRP1, and TRP2 were purchased from Santa Cruz Biotechnology (Dallas, TX, USA), MITF was purchased from Cell Signaling Technology Inc. (Danvers, MA, USA), and α-tubulin was purchased from Sigma-Aldrich (St. Louis, Missouri, USA). Human and mouse eumelanin enzyme-linked immunosorbent assay (ELISA) kits were purchased from MyBioSource (San Diego, California). Human and mouse TRP2 small interfering RNAs (siRNAs) were purchased from Santa Cruz Biotechnology. Steroidal saponins, including ACC tested in the present work, were isolated from the roots of *A. cochinchinensis* in our previous study^15^.

### Cell culture and treatment

B16F10 melanoma cells were purchased from the Korean Cell Line Bank and human melanoma MNT1 cells were kindly provided by Prof. Ai Young Lee (Department of Dermatology, DonggukUniversity Seoul, Graduate School of Medicine, Goyang, Republic of Korea). Both cells were cultured in DMEM supplemented with 10% FBS, 1% penicillin/ streptomycin at 37 °C in a 5% CO_2_ incubator. B16F10 cells were treated with 200 nM α-MSH, with or without ACC (5 or 10 μM), while MNT1 cells were treated with or without ACC (5 or 10 μM) in DMEM containing 2% FBS for 48 and 72 h.

### Cell viability assay

B16F10 and MNT1 cells were seeded at 4 × 10^4^ cells per well in a 96-well plate and grown overnight with or without ACC (5 or 10 μM). After 48 and 72 h, 0.1% 3-(4,5-dimethylthiazol-2-yl)-2-5-diphenyltrazolium bromide (tetrazolium salt) was added and cells were incubated for 1 h. Then, dimethyl sulfoxide was added to the stained cells and absorbance was measured at 570 nm wavelength using a microplate reader (VersaMax microplate reader).

### Melanin content assay

B16F10 and MNT1 cells were seeded at 4 × 10^4^ cells per well in a 48-well plate and incubated for 24 h at 37 °C in a 5% CO_2_ incubator. Cells were treated with 5 or 10 µM ACC, and the melanin content assay was performed after 48 and 72 h. Cells were washed twice with PBS, 200 μL of 1 *N*-sodium hydroxide was added, and cells were incubated at 60 °C for 1 h. The change in absorbance was measured at 405 nm wavelength using a microplate reader.

### Western blotting analysis

First, 70–80% confluent B16F10 and MNT1 cells were treated with 5 or 10 µM ACC for 48 h and 72 h. The cells were lysed with Pro-prep™ solution (iNtRON Biotechnology, Seoul, South Korea) and centrifuged at 10,000×*g* for 30 min at 4 °C. The supernatant was collected and protein concentration was estimated using the Bio-Rad Bradford assay. Whole proteins (20 μg) were separated according to size via sodium dodecyl sulfate–polyacrylamide gel electrophoresis. Proteins were then transferred to polyvinylidene fluoride membranes, blocked using 5% skim milk in Tris-buffered saline with 0.05% Tween-20 (TBST), and incubated overnight with tyrosinase, TRP1, TRP2, MITF, and α-tubulin (1:1000) diluted with 5% bovine serum albumin in TBST at 4 ℃. Western immunoreactive bands were visualized using Immobilon Western Chemiluminescent HRP Substrate (MERCK) and quantified using ChemiDoc (BioRad Laboratories, Hercules, CA, USA).

### Tyrosinase activity assay

To determine the tyrosinase activity of B16F10 and MNT1 cells, cells were lysed with Pro-prep™ solution as described above, and the total protein concentration in the cell lysates was estimated using the Bio-Rad Bradford assay. For tyrosinase activity, 1 mL of reaction mixture contained 50 mM phosphate buffer (pH 6.8), 2 mM L-DOPA, 1 mg of supernatant protein with/without 5 or 10 µM ACC, or 10 or 100 µM kojic acid (KA). After 15 min of reaction at 37 °C, dopachrome formation was determined by measuring the absorbance at 470 nm.

### Eumelanin and pheomelanin content assay

To measure eumelanin production, B16F10 and MNT1 cells were seeded in a 6-well plate. Overnight-grown cells were treated with or without 5 or 10 µM ACC. After 48 and 72 h, conditioned media of B16F10 and MNT1 cells were collected and stored at − 80 °C until further use. According to the manufacturer’s instructions, eumelanin and pheomelanin production was measured using eumelanin and pheomelanin ELISA kits (MyBioSource).

Furthermore, we analyzed pyrrole-2,3,5-tricarboxylic acid (PTCA) and thiazole-2,4,5-tricarboxylic acid (TTCA) for measuring the ratio of pheomelanin/eumelanin by using HPLC systemas described previous study within minor modification^34^. B16F10 and MNT1 cells were seeded in 100Φ dishes. Overnight-grown cells were treated with 200 nM α-MSH, with or without ACC (5 or 10 μM). After 48 and 72 h, conditioned media of B16F10 and MNT1 cells were collected and lysed with 1 mol/L KOH. After homogenization, oxidation reaction was formed by using H_2_O_2_ (final concentration 1.5% v/v) for 1 day. After that remained peroxide was quenched by using 10% Na_2_SO_4_. The pH of samples was adjusted to 3.3 and then filtered with syringe filter (0.22 μm pore size, GVS Filter Technology, USA). Cell samples were analysed using a Waters HPLC system with photodiode array (PDA) detector. For the analysis of PTCA and TTCA, a Sepax HP-C18 column (250 × 4.6 mm, 5 µm) was used. The mobile phase consisted of methanol-20 mM potassium phosphate pH 3.3 [17:83 (v/v%)]. The flow rate was 0.6 mL/min and PDA detector was set at 225 and 275 nm for PTCA and TTCA.

### UVB irradiation

Human keratinocytes, HaCaT cells were obtained from Korean Cell Line Bank (Seoul, South Korea) and were cultured in DMEM media supplemented with 10% FBS, 1% penicillin/streptomycin at 37 °C in a 5% CO_2_ incubator. 70–80% confluent cells were washed with 1X PBS and were irradiated with 125 mJ/cm^2^ UVB using a UV irradiation system (BIO-LINK^®^ BLX-365; Vilber Lourmat, Collegien, France) with an emission wavelength of 315 nm. After UVB irradiation the cells were cultured in fresh DMEM media and were treated with or without ACC (5 and 10 μM). After 24 h the conditioned media (CM) were collected and then treated to MNT1 cells. Western blot, eumelanin and pheomelanin content were analysed as described above.

### Zebrafish experiments

Zebrafish embryos were obtained from Kyung Hee University and maintained in egg water containing 60 μg/mL sea salt (Sigma-Aldrich, St. Louis, MO, USA) in distilled water. 9 hfp, zebrafish embryos were arrayed in a 24-well plate (eight embryos per well) containing 2 mL egg water. The embryos were treated with or without 1 or 5 μMACC and 25 μM 1-phenyl-2-thiourea (PTU) and incubated in a 28.5 °C incubator. After 72 hfp, the embryos were mounted on 2% agarose and imaged using a IX7 microscope (Olympus, Japan). The present study adhered to standard zebrafish protocols and was conducted with the approval of the Animal Care and Use Committee of Kyung Hee University [KHUASP(SE)-15-10], (Seoul, Korea) in accordance with ethical guidelines for animal research. All experimental procedures involving zebrafish were carried out in compliance with these established protocols and also follow the ARRIVE guidelines.

### Statistical analysis

Data are expressed as the mean ± standard deviation (SD) of the mean of a minimum of three independent experiments. Statistical comparisons were made between the control and treatment groups using one-way analysis of variance with Tukey’s test for multiple comparisons using GraphPad Prism 5.0 (GraphPad Software Inc., San Diego, CA, USA). *p* < 0.05 was considered to be statistically significant.

### Supplementary Information

Supplementary Information.

## Data Availability

All data generated or analyzed in this study are included in this article and its supplementary data files.
