# Supplementary material for: Aspacochioside C from Asparagus cochinchinensis attenuates eumelanin synthesis via inhibition of TRP2 expression
Source: Sci Rep. 2023 Sep 8;13:14831. doi: 10.1038/s41598-023-41248-5 (PMC10491620; doi:10.1038/s41598-023-41248-5)

**Supplementary Data**

**Aspacochioside C from *Asparagus cochinchinensis* attenuates eumelanin synthesis via inhibition of TRP2 expression**

Silvia Yunmam^a,b†^, Hae Ran Lee^a,†^, Seong Min Hong^a^, Ji-Young Kim^c^, Tong Ho Kang^d^, Ai Young Lee^e^, Dae Sik Jang^c,*^, Sun Yeou Kim^a,f,*^

*Reverse transcription-polymerase chain reaction (RT-PCR)*

B16F10 and MNT1 cells were seeded in a 12-well plate and treated with 5 or 10 µM aspacochioside C (ACC). RNA was extracted using TRIzol reagent (Invitrogen). Next, 500 ng of cDNA was prepared using the Takara PrimeScript RT reagent (Takara, Tokyo, Japan), following the manufacturer’s instructions. Quantitative RT-PCR (qRT-PCR) was performed using TB Green Premix EX Taq (Takara, Tokyo, Japan). All reactions were performed in triplicate with an initial denaturation at 95 ^o^C for 30 s, followed by 40 cycles at 95 ^o^C for 5 s and 60 ^o^C for 34 s. α-tubulin expression was used as the internal RNA standard for all data analyzed. The primers used for qRT-PCR in this study are listed in Table 1.

**Table 1: Primer sequences used for quantitative reverse transcription-polymerase chain reaction (qRT-PCR)**

| Gene Sequence |
| --- |
| *GAPDH* (m) fwd 5′-TGAAGGTCGGTGTGAACGGATTTGGC-3′  rvs 5′-CATGTAGGCCATGAGGTCCACCAC-3′  *TRP-1* (m) fwd 5′-GCTGCAGGAGCCTTCTTTCTC-3′  rvs 5′-AAGACGCTGCACTGCTGGTCT-3′  *TRP-2* (m) fwd 5′-GCTCCAAGTGGCTGTAGACC-3′  rvs 5′-AATGCAGTGGCTTGGAAATC-3′  *GAPDH* (h) fwd 5′-ACATCGCRCAGACACCATG-3′  rvs 5′-GGATCTTCACCATGCCATCTG-3′  *TRP-1* (h) fwd 5′-TCTCTGGGCTGTATCTTCTTCC-3′  rvs 5′-GTCTGGGCAACACATACCACT-3′  *TRP-2* (h) fwd 5′-CTTGGGCTGCAAAATCCTGC-3′  rvs 5′-CAGCACTCCTTGTTCACTAGG-3′ |

**
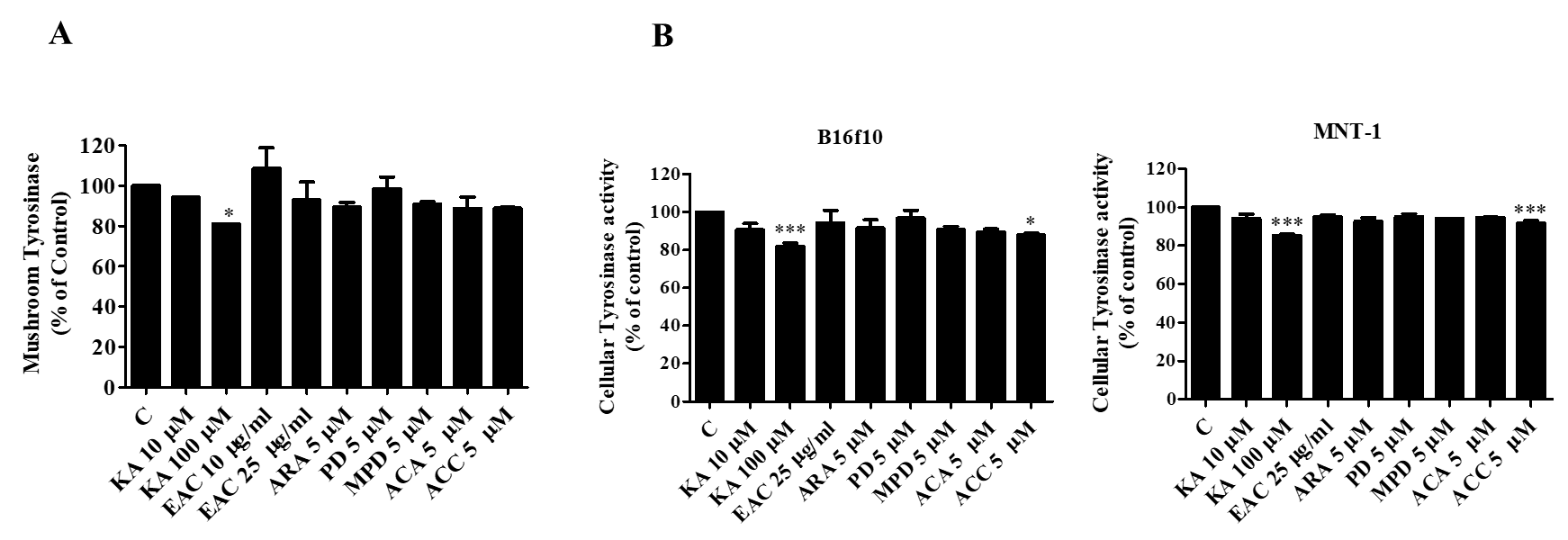
**

**Supplementary Fig. 1: Effects of the hot water extract of *Aspergillus cochinchinensis* and five steroidal saponins.** (A) Mushroom tyrosinase activity of the hot water extract of *A. cochinchinensis* and five steroidal saponins. (B) Cellular tyrosinase activity of the hot water extract of *A. cochinchinensis* and five steroidal saponins in B16F10 and MNT1 cells. EAC: hot water extract of the roots of *A. cochinchinensis*, ARA: asparacochioside A, PD: protodioscin, MPD: methyl protodioscin, ACA: aspacochioside A, and ACC: aspacochioside C. Kojic acid was used as a positive control. The values represent the mean ± standard deviation (SD) of three independent experiments. *p < 0.05, ***p < 0.001 vs. C

**
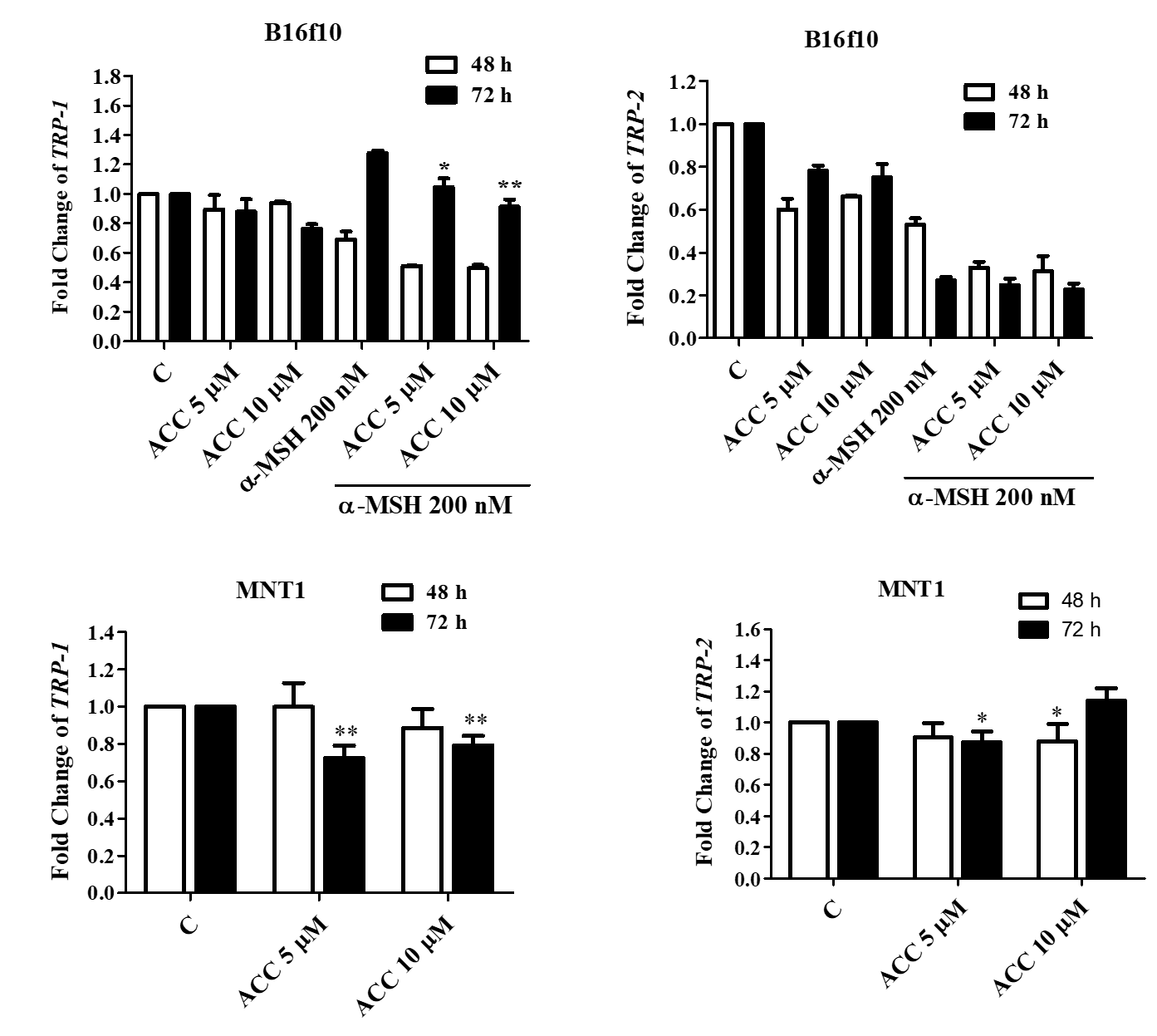
**

**Supplementary Fig. 2: Effects of aspacochioside C (ACC) ontyrosinase-related protein (TRP)-1 and TRP2 mRNA expression levels in B16F10 and MNT1 cells at 48 and 72 h.** The values represent the mean ± SD of three independent experiments. *p < 0.05, **p < 0.01 vs. C

**
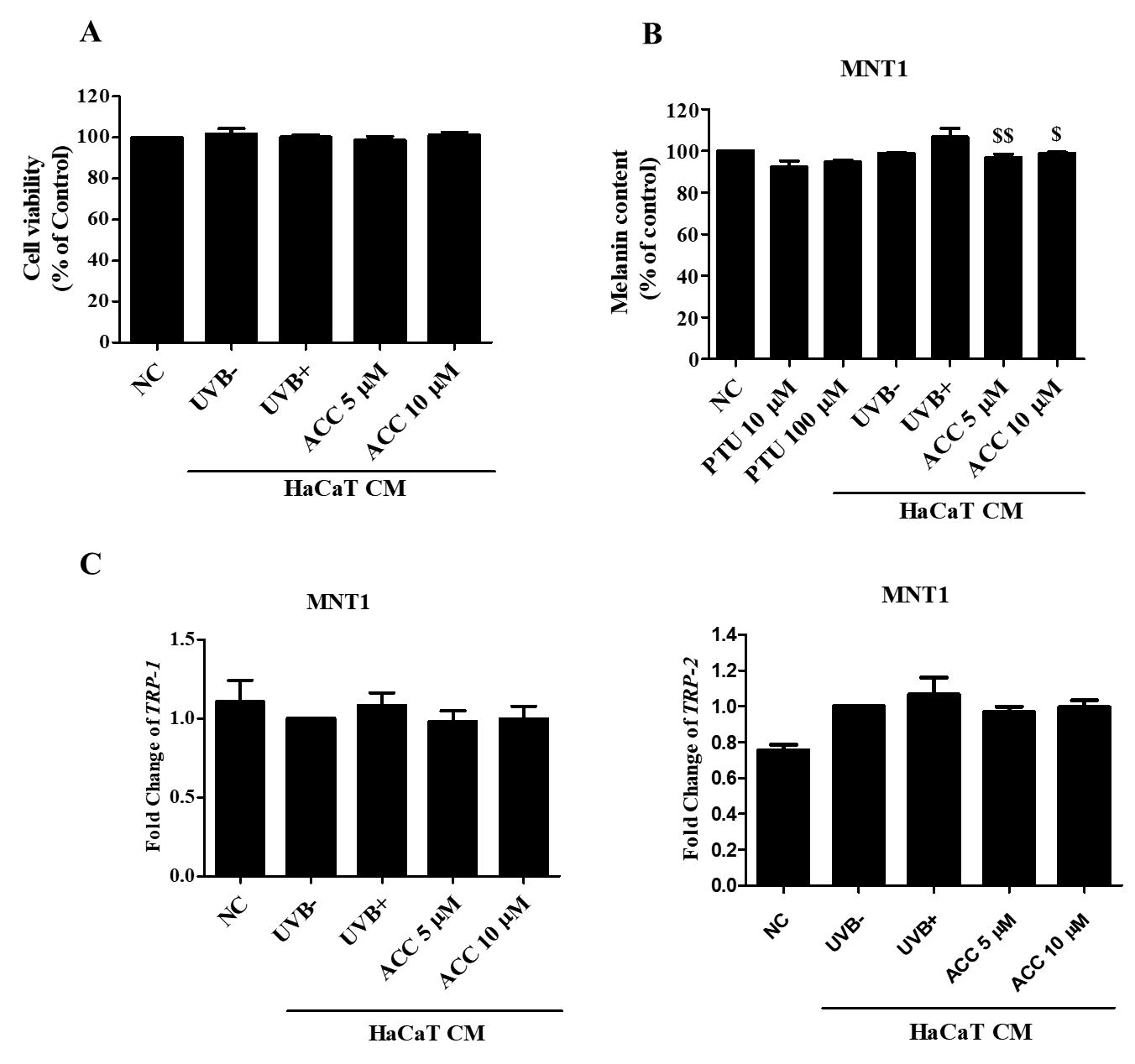
**

**Supplementary Fig. 3: Effect of aspacochioside C (ACC) on UVB-induced MNT1 cells.** HaCaT cells were irradiated with 125 mJ/cm^2^ UVB and treated with or without ACC (5 or 10 μM). After 24 h, the conditioned media (CM) of HaCaT cells were collected and used to treat MNT1 cells. (A) Effect of ACC on UVB-induced cell toxicity. (B) Effect of ACC on UVB-induced melanin production. (C) Effect of ACC on TRP1 and TRP2 mRNA expression levels in UVB-induced MNT1 cells. NC: MNT1 cells grown in 2% FBS containing Dulbecco’s modified Eagle’s medium (DMEM), UVB^-^: MNT1 cells grown in HaCaT CM, UVB^+^: MNT1 cells grown in UVB irradiated HaCaT CM. The values represent the mean ± SD of three independent experiments. ^$^p < 0.05, ^$$^p < 0.001 vs. UVB^-^


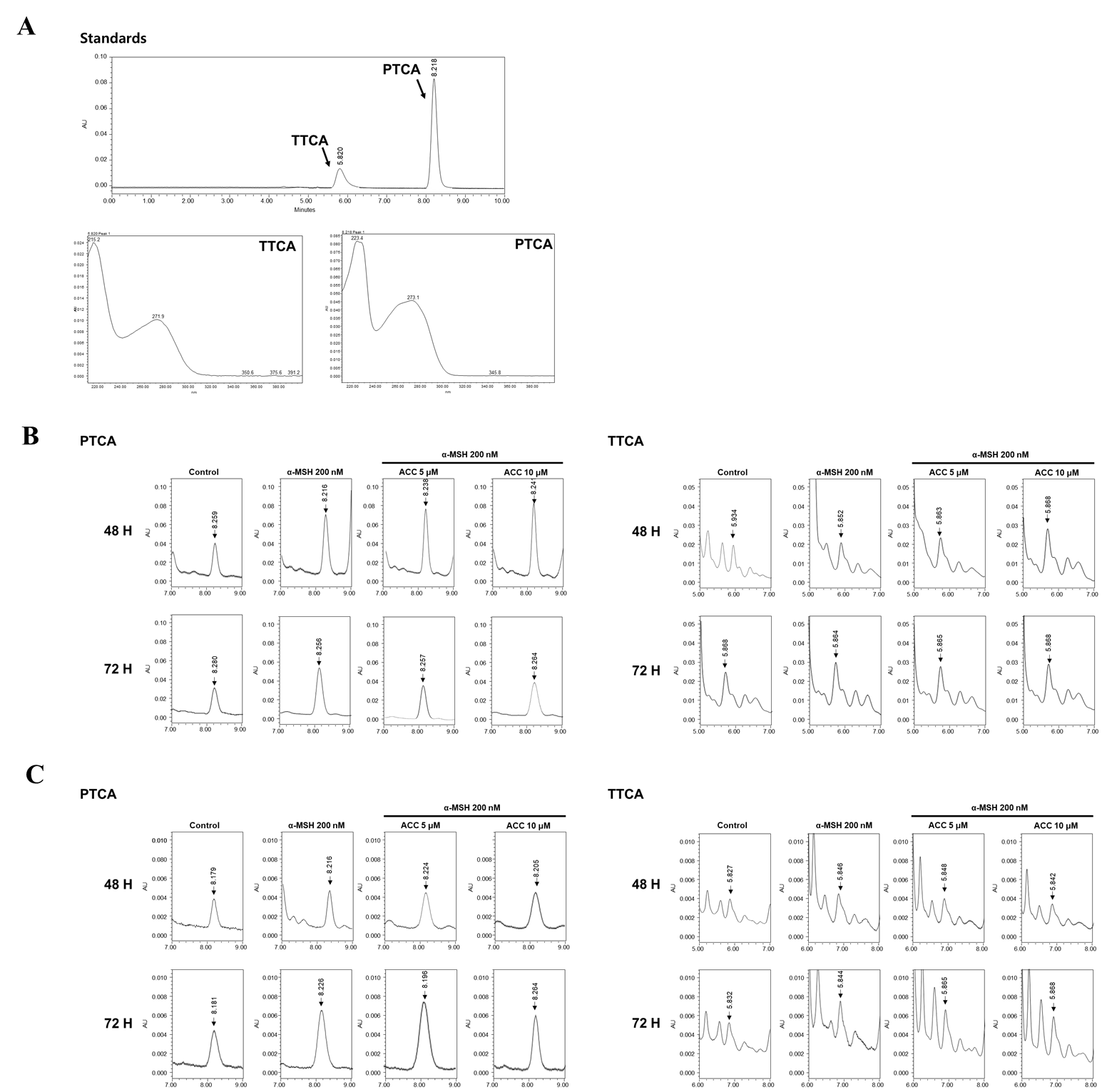


**Supplementary Fig. 4: Effect of aspacochioside C (ACC) on ratio of pheomelanin (TTCA)/eumelanin (PTCA) production.** Cells were treated with ACC as described. The ratio of pheomelanin (TTCA)/eumelanin (PTCA) was measured using HPLC analysis. (A) Chromatograms of standard including PTCA and TTCA. (B) Chromatograms of PTCA and TTCA in α-MSH-induced B16F10 cells. (C) Chromatograms of PTCA and TTCA in α-MSH-inducedMNT1 cells.

**Western Blot original blot**


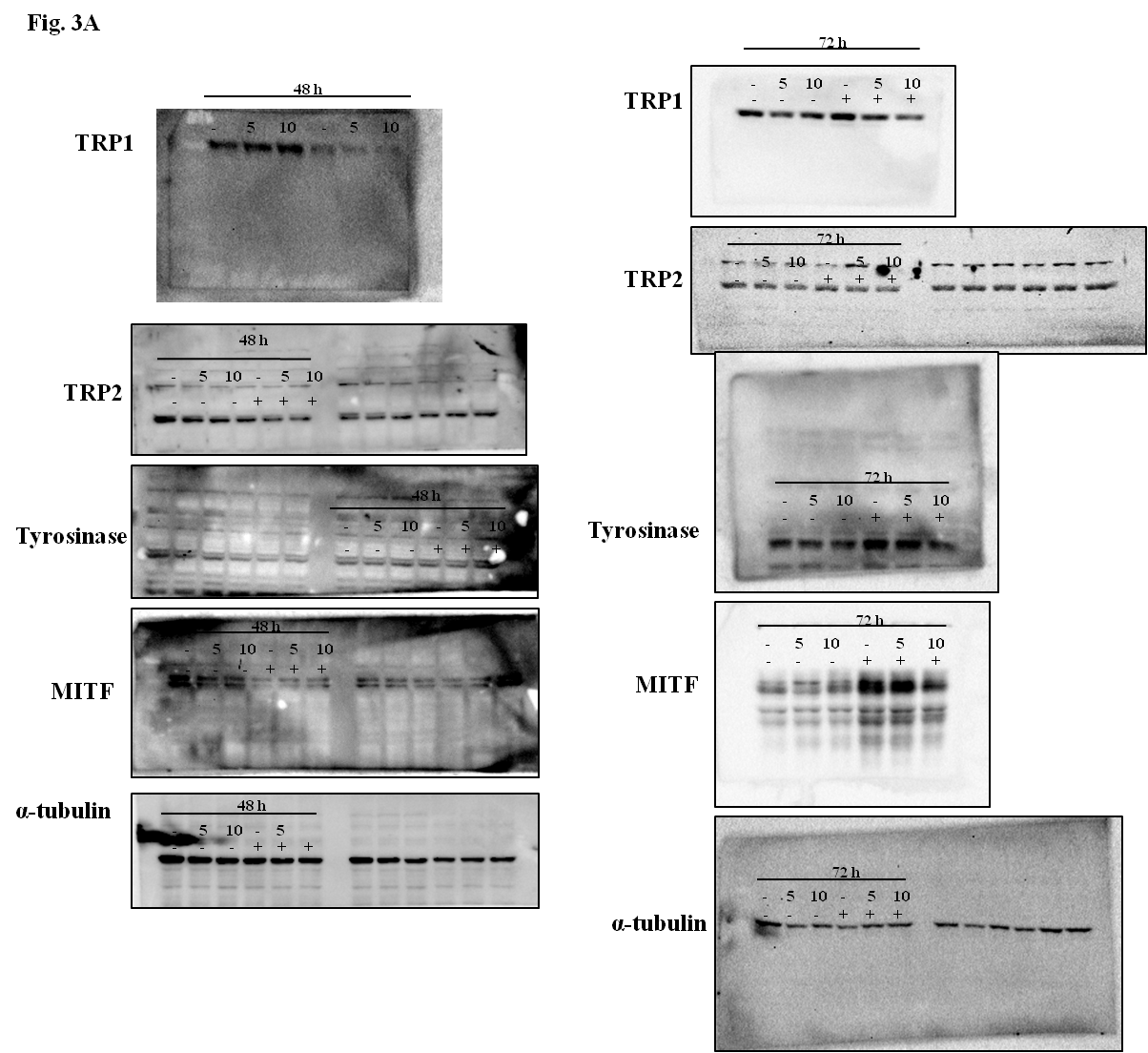


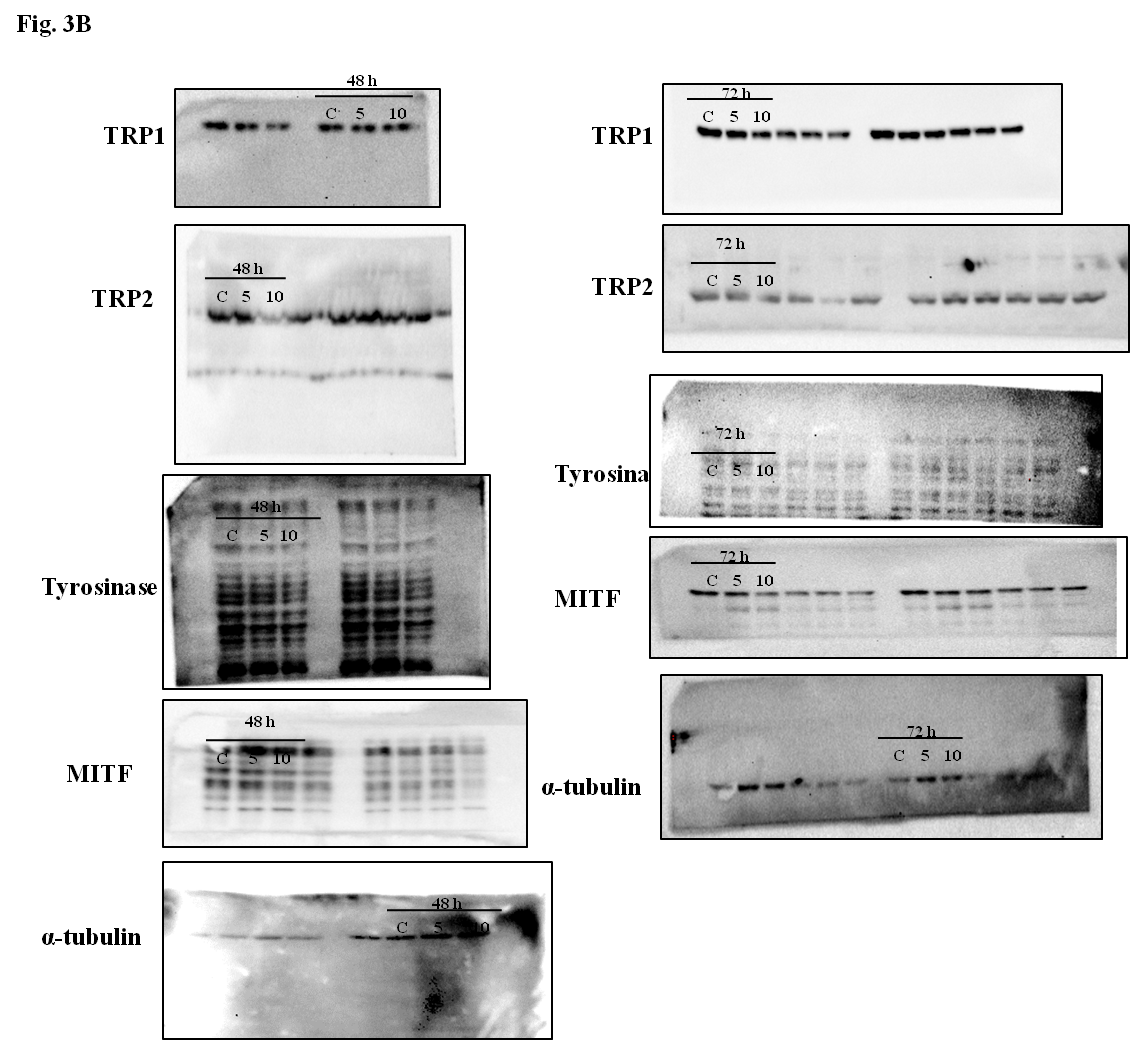


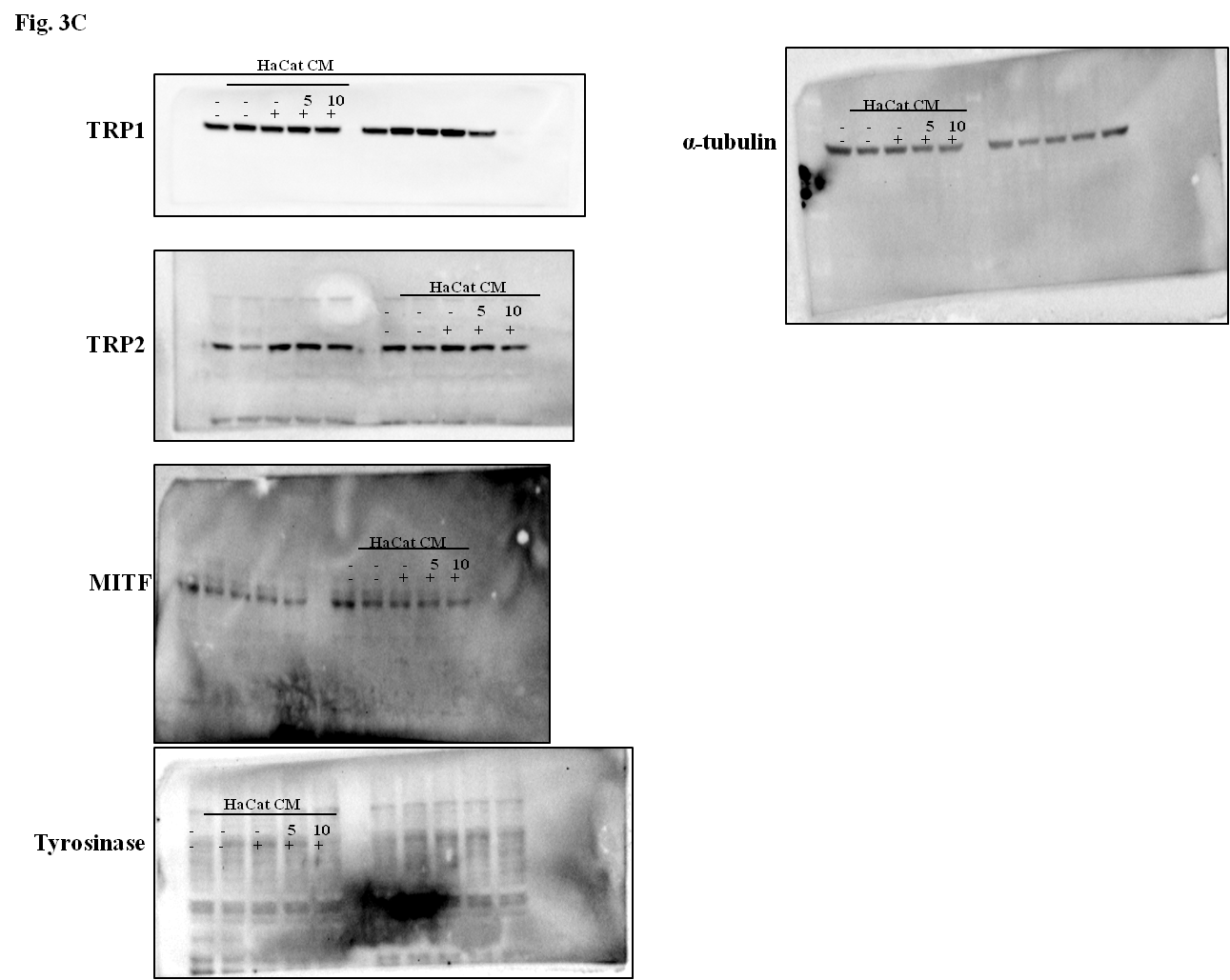

Supplement: Supplementary file 1 — Supplementary Information. [file 41598_2023_41248_MOESM1_ESM.docx]
